# ARS2, a Cofactor of CBC, Promotes Meiotic Silencing by Unpaired DNA

**DOI:** 10.3390/epigenomes10010006

**Published:** 2026-01-21

**Authors:** Michael M. Vierling, Victor T. Sy, Logan M. Decker, Hua Xiao, Justine N. Hemaya, Patrick K. T. Shiu

**Affiliations:** Division of Biological Sciences, University of Missouri, Columbia, MO 65211, USA

**Keywords:** cap-binding proteins (CBPs), genome defense, meiotic silencing by unpaired DNA (MSUD), *Neurospora crassa*, RNA interference (RNAi)

## Abstract

The presence of an extra DNA segment in a genome could indicate a transposon or another repetitive element on the move. In *Neurospora crassa*, a surveillance mechanism called meiotic silencing by unpaired DNA (MSUD) is maintained to monitor these selfish elements. MSUD utilizes common RNA interference (RNAi) factors, including the SMS-2 Argonaute, to target mRNAs from genes lacking a pairing partner during meiosis. In eukaryotes, an mRNA transcript is typically bound at the 5′ cap by the cap-binding complex (CBC), which assists in its nuclear export. Previously, we discovered that CBC and its interactor NCBP3 mediate MSUD, possibly by guiding the perinuclear SMS-2 to effectively recognize exported mRNAs. Here, we report that ARS2, a CBC cofactor, is involved in MSUD. ARS2 interacts with both CBC and NCBP3, and it may help bring them together. In addition to its role in silencing, ARS2 also contributes to vegetative growth and sexual sporulation.

## 1. Introduction

*Neurospora crassa* is a haploid fungus that grows as a network of filamentous cells (hyphae) and has a distinct sexual cycle [1]. To keep selfish genetic elements at bay, *Neurospora* maintains several genome surveillance mechanisms to suppress their gene expression [2]. One of these is known as meiotic silencing by unpaired DNA (MSUD) [3,4,5]. In this mechanism, a gene not having a pairing partner during meiosis is seen as a potential threat and is subject to silencing. For example, MSUD can recognize an unpaired transposon and target its sequence during the sexual cycle [6].

MSUD begins in the nucleus when the direct pairing of homologous double-stranded DNAs is inspected. This pairing process may rely on REC8 (meiotic kleisin) and SAD-6 (chromatin remodeler) [7,8]. Once an unpaired gene is detected, an aberrant RNA (aRNA) is transcribed and exported to the perinuclear region. SAD-1 (RNA-directed RNA polymerase), assisted by SAD-3 (helicase), changes the aRNA into a double-stranded RNA (dsRNA) [9,10]. The dsRNA is cut up by DCL-1 (Dicer) into small interfering RNAs (siRNAs), which are then made into single strands by QIP (exonuclease) [11,12,13]. The single-stranded siRNAs can subsequently guide SMS-2 (Argonaute) to seek out homologous mRNAs for destruction [14]. SAD-2, a scaffold protein, is in charge of bringing SAD-1 and others to the perinuclear region [15,16,17].

In addition to the aforementioned proteins, the nuclear cap-binding complex (CBC) also plays a role in meiotic silencing [18]. In eukaryotes, CBC binds to the 5′ cap of RNA polymerase II transcripts and facilitates a variety of processing events, including pre-mRNA processing, nuclear export, and others [19,20]. CBC is made up of nuclear cap-binding proteins NCBP1 and NCBP2 (also known as CBP80 and CBP20, respectively). According to our MSUD model, CBC binds to NCBP3 (SAD-8), and the resulting complex helps deliver an mRNA to the SMS-2 Argonaute in the perinuclear region [21]. Since ARS2 (arsenite resistance protein 2) is thought to recruit NCBP3 to CBC in mammals [22,23], we set out to explore if it also influences meiotic silencing in *Neurospora*.

## 2. Materials and Methods

### 2.1. Fungal Methods and Genotypes

Standard fungal procedures, according to the *Neurospora* protocol guide, were used in this study (https://www.fgsc.net/Neurospora/NeurosporaProtocolGuide.htm; accessed on 26 August 2025). Strain names and genotypes are listed in Table 1. Progenitor strains, including those from the knockout library [24], were acquired from the Fungal Genetics Stock Center (FGSC) [25]. The sequence of *ars2* (*ncu02800-t26_1*) was obtained from FungiDB [26]. Fungal isolates were grown and maintained on Vogel’s medium [27]. Crosses were performed on SC (synthetic crossing) medium [28].

### 2.2. Assays for Growth, Sexual Development, and MSUD Suppression

Linear growth rates of fungal strains were recorded at room temperature with race tubes [29]. Quantification of ascospore (sexual spore) production was carried out as per Hammond et al. [10]. Analysis of MSUD suppression was essentially as described by Xiao et al. [30]. Briefly, perithecia were grown in a well of a microtiter plate, and the resulting ascospores were shot onto the plate lid and later collected for the microscopic examination of their phenotypes. For the aforementioned assays, the *p*-values were calculated using the two-tailed Student’s *t*-test.

### 2.3. Strain Construction and Confirmation

The mCherry tagging vector for *ars2* was constructed using double-joint polymerase chain reaction (DJ-PCR) [7]. *Neurospora* transformation by electroporation of conidia (asexual spores) was as described by Margolin et al. [31]. For genotype confirmation, genomic DNA was purified from conidia [32] or hyphae (DNeasy Plant Mini Kit; QIAGEN, Germantown, MD, USA). For screening and validation of genotypes, PCR was performed using the GoTaq Green Master Mix (Promega, Madison, WI, USA) or the Expand Long Range dNTPack (Roche Diagnostics, Indianapolis, IN, USA). Sanger DNA sequencing was conducted by the University of Missouri (MU) Genomics Technology Core. Primers for this study are listed in Supplementary Table S1.

### 2.4. Bimolecular Fluorescence Complementation (BiFC)

BiFC is an in vivo protein–protein interaction assay that relies on the reconstitution of a fluorophore [33,34]. In this assay, the N-terminal half of the yellow fluorescent protein (YFPN) is tagged to a protein of interest, while the C-terminal half (YFPC) is tagged to another. A positive interaction between the two proteins of interest is indicated by the restored yellow fluorescence. YFPN and YFPC were tagged to various MSUD proteins using an established method [35].

### 2.5. Photography and Microscopy

Z-stack images of protoperithecia (female structures) were captured using an M205 FA stereomicroscope equipped with a DFC345 FX camera (Leica Microsystems, Deerfield, IL, USA). For perithecia (fruiting bodies), pictures were taken by an Apple iPhone 5 (fitted with a Magnifi photoadapter; Arcturus Labs, Lawrence, KS, USA) on a VanGuard 1231CM microscope (VEE GEE Scientific, Vernon Hills, IL, USA). Photographs of asci (spore sacs) were obtained from a BX45 microscope equipped with a DP74 camera (Olympus, Center Valley, PA, USA). For fluorescence microscopy, preparation and viewing of asci were as previously described [11,13], with the employment of a Leica TCS SP8 system at the MU Advanced Light Microscopy Core.

## 3. Results

### 3.1. ARS2 Plays a Role in MSUD

**Figure 1:**
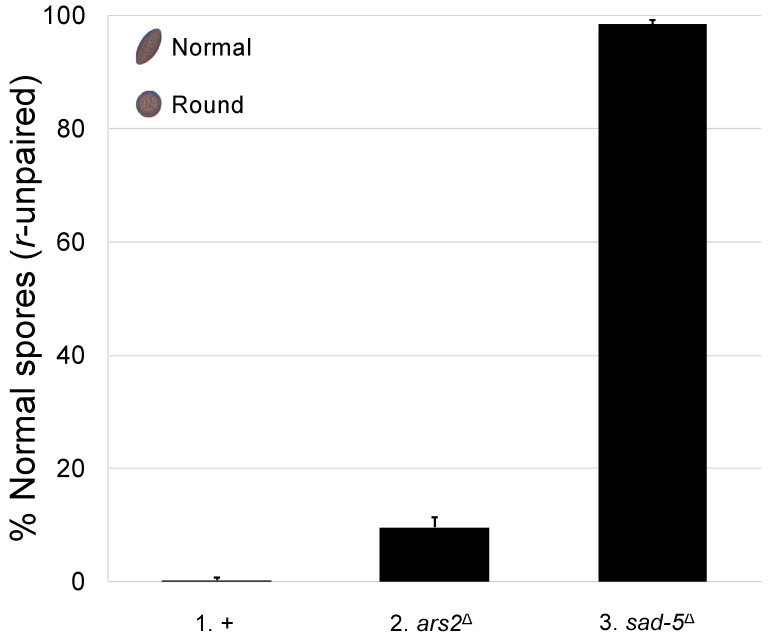
The loss of ARS2 is associated with a reduction in MSUD activity. Normal *Neurospora* ascospores are of American football shape (see upper-left insert). In this experiment, crosses with an unpaired *round spore* gene (i.e., *r*^+^ × *r*^∆^) were examined. When MSUD is proficient, the unpaired *r*^+^ gene is silenced, and most progeny are round (i.e., 0.27% football; cross 1). However, if both parents are missing SAD-5 (an essential MSUD protein) [37], silencing becomes deficient, and most progeny are normal (i.e., 98.4% football; cross 3). When the cross is lacking ARS2, only a modest increase in the number of normal progeny is observed (i.e., 9.6% football, cross 2; *p* < 0.001), suggesting that this protein plays a supplementary role in silencing. Hereafter, an error bar indicates the standard deviation among three replicates. +, wild type at pertinent loci. Crosses: (1) F2-30 × P3-08. (2) F6-26 × P20-49. (3) F5-36 × P17-70.

We have previously shown that CBC and NCBP3 mediate MSUD in *Neurospora* [18,21]. Since ARS2 interacts with these factors in other organisms [22,36], we asked whether it is also involved in meiotic silencing. In a typical *Neurospora* cross, American football-shaped ascospores are produced. On the other hand, if the *round spore* gene is unpaired (i.e., *r*^+^ × *r*^∆^), it will be meiotically silenced, and the progeny will be mostly round (i.e., 0.27% football; Figure 1, cross 1). This abnormal phenotype can be mitigated if the cross is lacking an MSUD protein. As seen in Figure 1 (cross 2), the deletion of *ars2* in both parents leads to 9.6% of the progeny showing the unsilenced phenotype, suggesting that ARS2 plays a role in meiotic silencing.

### 3.2. ARS2 Is Involved in Vegetative Growth

**Figure 2:**
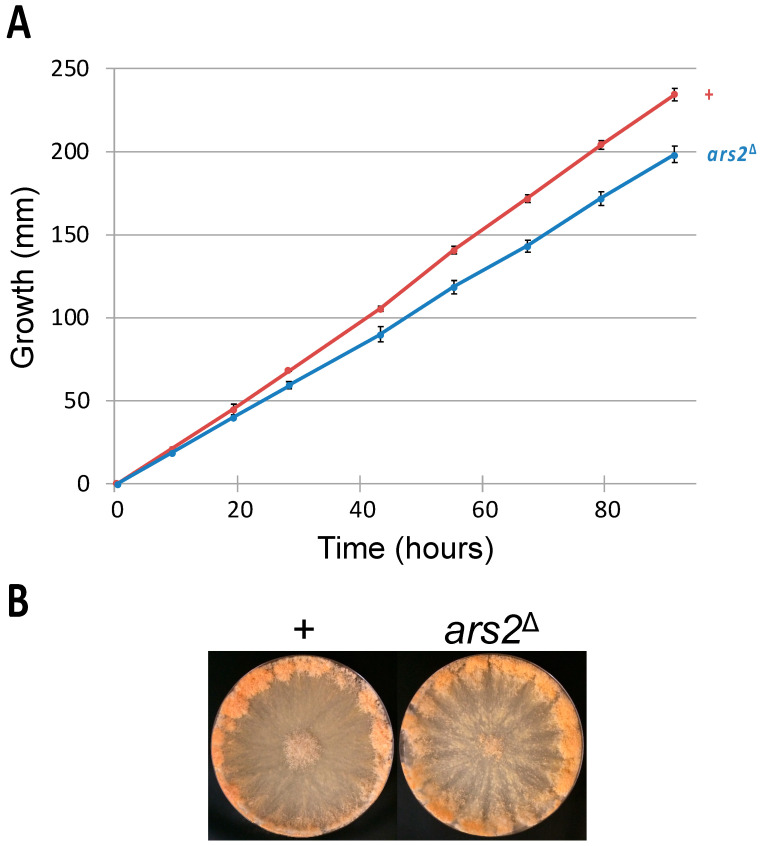
Vegetative traits of an *ars2* mutant. (**A**) An *ars2*^∆^ mutant has a significantly slower linear growth rate as compared to that of a wild-type strain (198 versus 234 mm at the 91 h mark; *p* < 0.001). (**B**) The *ars2* deletion does not affect the proficiency of conidiation. Strains: P3-08 and P20-50.

A loss-of-function mutation in *ARS2* (or its homolog) is lethal in *Schizosaccharomyces pombe* [38], *Arabidopsis thaliana* [39], *Drosophila melanogaster* [40], *Danio rerio* [41], and *Mus musculus* [42]. In *Neurospora*, while the *ars2* gene is nonessential for the vegetative stage, its deletion is linked to a slower linear growth rate: an *ars2*^∆^ mutant covers only 85% of the distance attained by a wild-type strain at the end of a race-tube assay (Figure 2A).

In *Arabidopsis*, a nonlethal mutation in *SERRATE* (an *ARS2* homolog) is associated with various vegetative defects, e.g., reduced rosette leaf production [43]. In *Neurospora*, production of conidia appears to be proficient in an *ars2*^∆^ mutant (Figure 2B).

### 3.3. Mutation in ars2 Affects Sexual Development

**Figure 3:**
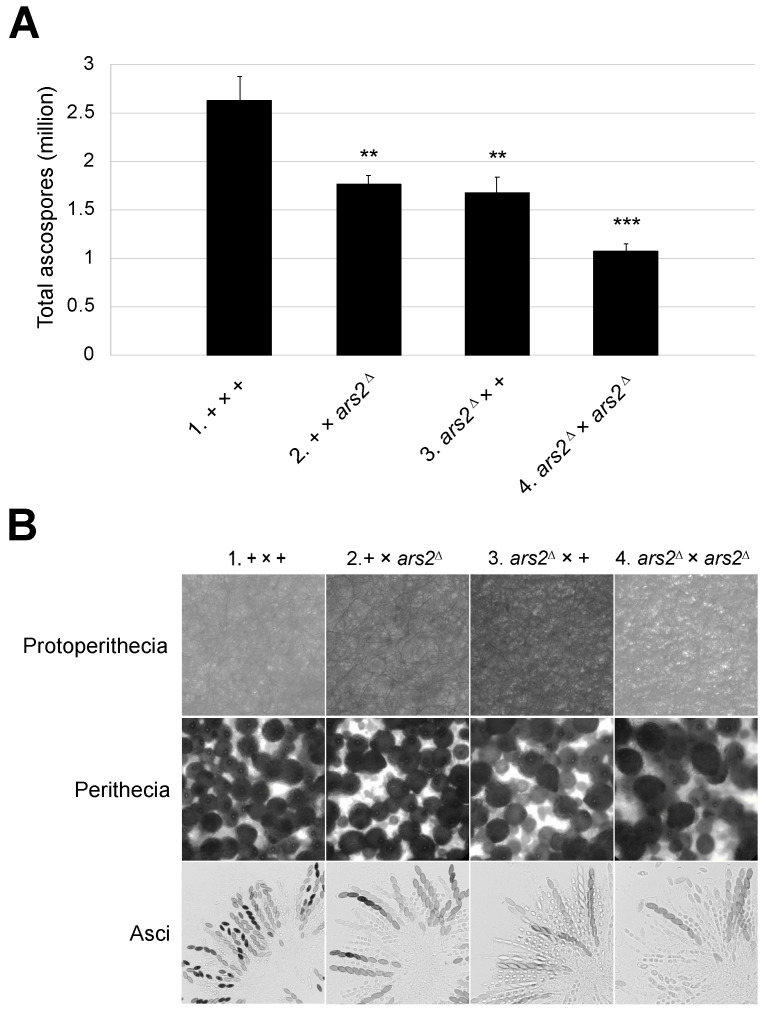
*ars2*^∆^ crosses produce fewer progeny. (**A**) The deletion of *ars2* leads to a marked reduction in ascospore production. A heterozygous cross with the *ars2* deletion in either the male or female parent produces only 67% (1.76 million; cross 2) or 63% (1.67 million; cross 3) of the normal number of ascospores (2.63 million; cross 1), respectively. A cross in an *ars2*-null background produces only 41% (1.08 million; cross 4) of the normal amount. For + versus mutant cross, ** indicates *p* < 0.01 and *** indicates *p* < 0.001. (**B**) While *ars2*^∆^ crosses appear proficient in protoperithecial and perithecial development, they have more aborted asci as compared to a normal cross. Crosses: (1) F2-01 × P3-08. (2) F2-01 × P20-50. (3) F9-09 × P3-08. (4) F9-09 × P20-50.

Many characterized MSUD proteins are essential for sexual sporulation [4,10,11,13,15,44]. As for *ars2*, when both parents are missing this gene, only 41% of the normal amount of ascospores are produced (Figure 3A, cross 4). Crosses heterozygous for *ars2*^∆^ fare somewhat better, achieving 63–67% of the normal ascospore production (Figure 3A, crosses 2 and 3). Upon examination of the mutant perithecia homozygous or heterozygous for *ars2*^∆^, we observed an increase in ascus abortions, suggesting that ARS2 is important for normal ascus development (Figure 3B).

### 3.4. ARS2 Is Associated with CBC

**Figure 4:**
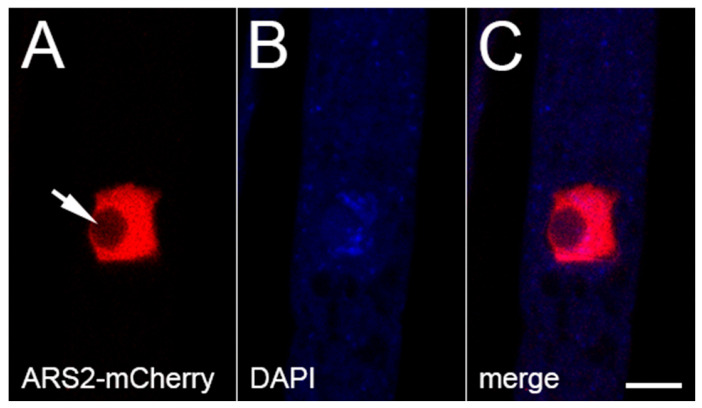
Subcellular localization of ARS2. ARS2 is predominantly found in the nucleus (excluding the nucleolus; arrow). Micrographs illustrate a prophase ascus expressing *ars2-mCherry* (P27-40 × P27-41). The chromatin was stained with DAPI. Bar, 5 µm.

**Figure 5:**
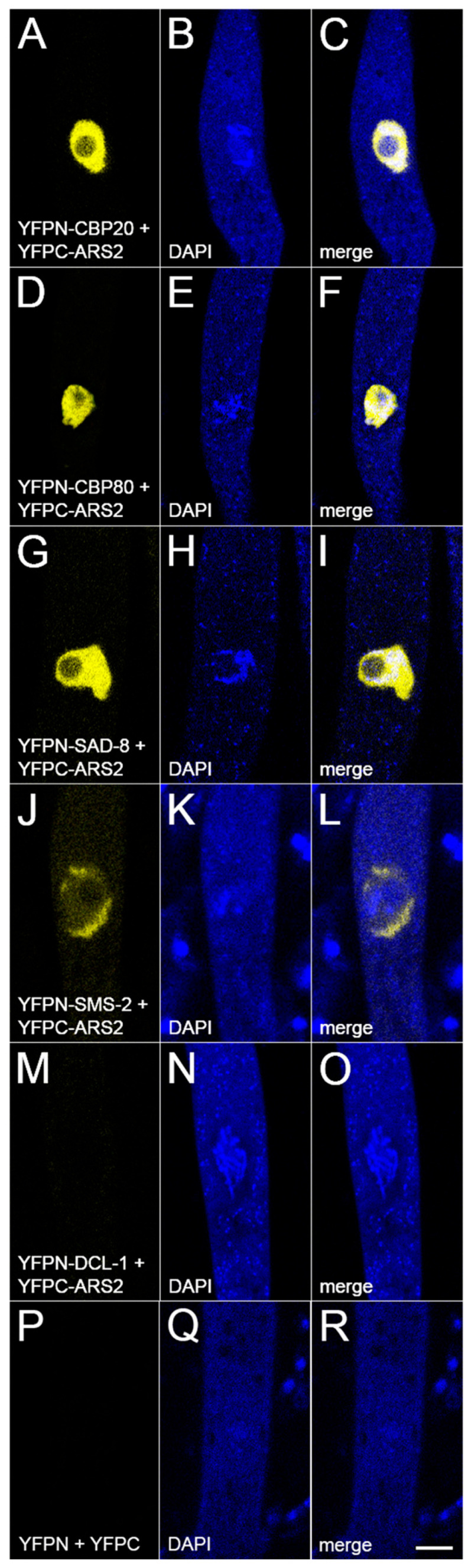
ARS2 interacts with NCBP1/2/3 and SMS-2. In a BiFC assay, the yellow fluorescent protein (YFP) is reconstituted if two tagged proteins interact. (**A**–**F**) ARS2 interacts with CBP20 (NCBP2) and CBP80 (NCBP1), both components of CBC. (**G**–**I**) ARS2 interacts with SAD-8 (NCBP3), another cap-binding protein. (**J**–**O**) ARS2 interacts with the SMS-2 Argonaute, but not the DCL-1 Dicer, in the perinuclear region. (**P**–**R**) Negative control. Micrographs illustrate prophase asci expressing (**A**–**C**) *yfpn-cbp20* and *yfpc-ars2* (P27-42 × P27-43), (**D**–**F**) *yfpn-cbp80* and *yfpc-ars2* (P30-23 × P30-24), (**G**–**I**) *yfpn-sad-8* and *yfpc-ars2* (P30-25 × P30-26), (**J**–**L**) *yfpn-sms-2* and *yfpc-ars2* (P27-48 × P27-49), (**M**–**O**) *yfpn-dcl-1* and *yfpc-ars2* (P31-04 × P31-05), and (**P**–**R**) *yfpn* and *yfpc* (P13-65 × P14-04). Bar, 5 µm.

ARS2 is a key cofactor of the nuclear CBC, and they form the CBC-ARS2 (CBCA) complex in fungi, plants, and animals [36,45]. Like its homologs, *Neurospora* ARS2 is predominantly nuclear (Figure 4). Using BiFC, we have obtained evidence that ARS2 is closely associated with both components of CBC (i.e., CBP20 and CBP80) in *Neurospora* (Figure 5A–F), supporting the notion that they form the CBCA complex in this fungus.

### 3.5. ARS2 Interacts with NCBP3 and the SMS-2 Argonaute

An mRNA is typically bound by CBC, which facilitates its nuclear export [19]. Our MSUD model suggests that the SMS-2 Argonaute may efficiently recognize exiting mRNAs by interacting with a complex containing CBC and NCBP3 [18,21]. One possible action of ARS2 is that it could help bring CBC and NCBP3 together, and the resulting complex interacts with SMS-2. In support of this hypothesis, our BiFC analysis shows that ARS2 indeed interacts with CBC (as noted above), NCBP3 (Figure 5G–I), and SMS-2 (Figure 5J–L).

## 4. Discussion

In eukaryotes, CBC binds to ARS2, which acts as a platform to recruit various RNA classifier factors that eventually decide the fate of the associated RNA [36]. One of the factors that ARS2 recruits is NCBP3, which is thought to promote mRNA export and translation (and possibly other RNA processing events) [22]. In this work, we have shown that ARS2 interacts with CBC in *Neurospora*, substantiating the idea that the former acts as a cofactor of the latter in this fungus. We have also shown that ARS2, like CBC and NCBP3, plays a role in MSUD. ARS2, as predicted, interacts with NCBP3 in *Neurospora*.

While ARS2 is essential for many eukaryotes, its significance in fungi is not uniform. For example, this protein is absent from *Saccharomyces cerevisiae* [36], essential for *S. pombe* [38], and not absolutely required for *Fusarium graminearum* [45]. Here, our results indicate that while *ars2* is not indispensable for *Neurospora*, its absence correlates with a slower linear growth and fewer sexual progeny.

**Figure 6:**
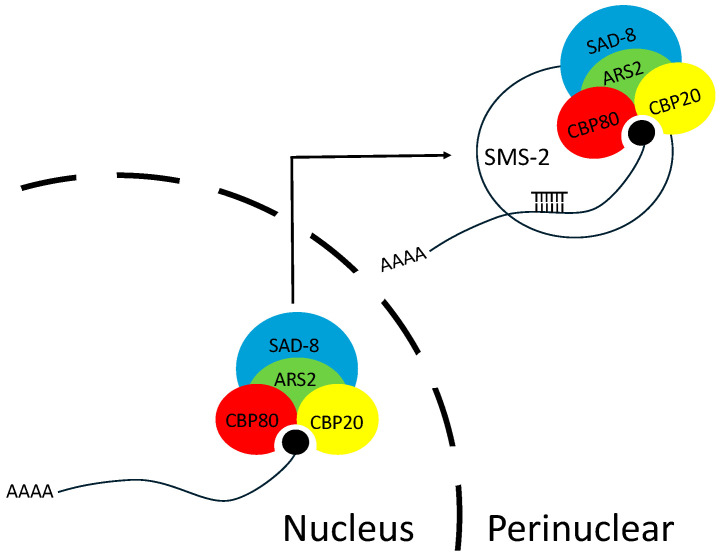
A model for the roles of nuclear cap-binding proteins and ARS2 in MSUD. In the perinuclear region, an siRNA-loaded Argonaute (SMS-2) can identify an exiting mRNA by interacting with NCBP1 (CBP80), NCBP2 (CBP20), and NCBP3 (SAD-8). ARS2 may help recruit NCBP3 to NCBP1/2 (also known as CBC), forming the optimal structure for Argonaute to recognize.

In MSUD, NCBP1/2/3-bound mRNAs are exported from the nucleus to the perinuclear region, where the SMS-2 (Argonaute) proteins await. This ternary cap-binding complex is presumably important for mRNA recognition by SMS-2 [18,21]. One possibility is that ARS2 helps recruit NCBP3 to CBC, forming the optimal structure for SMS-2 to detect (Figure 6). In the absence of ARS2, NCBP3 may still be able to bind to CBC, albeit with a lower efficiency. An alternative explanation is that ARS2 may stimulate the silencing machinery. In *Drosophila*, it has been suggested that Ars2 may bind to Dicer-2 and promote its enzymatic activity [46]. However, based on our BiFC assay, we do not have evidence showing that ARS2 interacts with the DCL-1 Dicer in *Neurospora* (Figure 5M–O).

During nuclear export, a myriad of molecules exit the nucleus, and it is incumbent on the perinuclear SMS-2 proteins to inspect each exported mRNA and ensure that no meiotic silencing targets can reach the translational machinery in the bulk cytoplasm. Thus far, our results have alluded to the importance of cap-associated proteins (i.e., CBC, NCBP3, and ARS2) in this process. Future work in this area will hopefully give us more insights into the mechanism by which mRNAs are effectively inspected and sorted out during MSUD.

## Figures and Tables

**Table 1:** *Neurospora* strains used in this study.

Strain	Genotype
F2-01	*fl A* (FGSC 4317)
F2-30	*rid r* ^Δ^ *::hph; fl A*
F5-36	*fl; sad-5* ^Δ^ *::hph a*
F6-26	*ars-2* ^Δ^ *::hph r* ^Δ^ *::hph; fl a*
F9-09	*ars2* ^Δ^ *;* *fl A*
P3-08	Oak Ridge wild type *a* (FGSC 2490)
P13-65	*rid his-3* ^+^ *::yfpn; mus-52* ^∆^ *::bar A*
P14-04	*rid his-3* ^+^ *::yfpc; mus-51* ^Δ^ *::bar a*
P17-70	*r* ^Δ^ *::hph; sad-5* ^Δ^ *::hph A*
P20-49	*ars2*^∆^*::hph A* (FGSC 15712)
P20-50	*ars2*^∆^*::hph a* (FGSC 16001)
P27-40	*rid ars2-mCherry::hph; mus-51* ^Δ^ *::bar A*
P27-41	*rid ars2-mCherry::hph; mus-51* ^Δ^ *::bar a*
P27-42	*rid his-3 yfpc-ars2::hph; yfpn-cbp20::nat1 mus-52* ^Δ^ *::bar a*
P27-43	*rid his-3 yfpc-ars2::hph; yfpn-cbp20::nat1; mus-51* ^Δ^ *::bar A*
P27-48	*rid yfpc-ars2::hph; yfpn-sms-2::hph a*
P27-49	*rid yfpc-ars2::hph; yfpn-sms-2::hph A*
P30-23	*rid yfpc-ars2::hph; yfpn-cbp80::hph a*
P30-24	*rid yfpc-ars2::hph; yfpn-cbp80::hph A*
P30-25	*rid yfpc-ars2::hph; yfpn-sad-8::nat1 A*
P30-26	*rid yfpc-ars2::hph; yfpn-sad-8::nat1 a*
P31-04	*rid yfpc-ars2::hph; mus-52* ^Δ^ *::bar; yfpn-dcl-1::nat1 a*
P31-05	*rid his-3 yfpc-ars2::hph; mus-52* ^Δ^ *::bar; yfpn-dcl-1::nat1 A*

## Data Availability

Supporting information is available in the Supplementary Materials.

## Supplementary Materials

The following supporting information can be downloaded at: https://www.mdpi.com/article/10.3390/epigenomes10010006/s1, Table S1: Primers for strain construction and confirmation.
