# Supplementary material for: ARS2, a Cofactor of CBC, Promotes Meiotic Silencing by Unpaired DNA
Source: Epigenomes. 2026 Jan 21;10(1):6. doi: 10.3390/epigenomes10010006 (PMC12922007; doi:10.3390/epigenomes10010006)
Supplement: Supplementary file 1 [file epigenomes-10-00006-s001.zip › epigenomes-3995446-supplementary.pdf]

Table S1. Primers for strain construction and confirmation.

Purpose	Primer	Sequence (5' to 3')
ars2-mCherry/yfpn construction	ARS2-A	CGTCTTTGAGAGTGACTCCATCC
	ARS2-GFP1	CAGCGCCTGCACCAGCTCCTGCCCCATAGTTGAGCTCTCCACCGTTTC
	ARS2-GFP2	CTCCTTCAATATCAGTTAACAAGGGCTCATGAAGGGAGGGATTGTG
	ARS2-B	CCCAACCCAACGGGATTA
	ARS2-C	CCCTTCCAAGAAGCGTAAGG
	ARS2-D	GGATGATGAGCGATCAAGGTAG
yfpc-ars2 construction	ARS2-E	TGCTGTTTGGAGGGAGATTG
	ARS2-NGFP1	GCAGCCTGAATGGCGAATGGACGCGCGTGGTCGGAACCGGATTAAG
	ARS2-NGFP2	CAGGAGCGGGTGCGGGTGCTGGAGCGATGGACTCGTCCTATCACTCTTA
	ARS2-F	GAAGACACGGCGAAGATACTC
	ARS2-G	CCTCTTGTGGACATCGCTTAG
	ARS2-H	TCATGTTCTTGGCCCTCTTC
yfpc-dcl-1 construction	DCL-1-E	CCCCGATAGTCCCATTACCCAATTC
	DCL-1-NGFP1	GCAGCCTGAATGGCGAATGGACGCGCAAGTGACAGACTGCTGTCGGGAATG
	DCL-1-NGFP2	CAGGAGCGGGTGCGGGTGCTGGAGCGATGGCCGTAGCCACTCGGCTACCCTT
	DCL-1-F	GACTTCTGCTGCAATGCTGGGTATG
	DCL-1-G	GCTGGACCCAAAGGGCTTTGTTATT
	DCL-1-H	TCGACCACGAACGTATTCAACAACC
yfpn-cbp20 construction	A-NTCbp20LF-LP	GAGAAGCTCCGGATTTTGGGCATC
	B-NTCbp20LF-RP	GCAGCCTGAATGGCGAATGGACGCGCGGTTACCTGGTAGTGAAGATGGATT
	C-NTCbp20RF-LP	CAGGAGCGGGTGCGGGTGCTGGAGCGATGTTGAACACTCGCCAAAGGAGC
	D-NTCbp20RF-RP	GATGCTGCCCAAGGACAAGTACAC
	E-NTCbp20-Nest-LP	AGTAGCTTGTGTTGAGGTCCTGCT
	F-NTCbp20-Nest-RP	GGCATTCCTTTTCCAGAGCTTCCC
yfpn-cbp80 construction	A-NT4187LF-LP	TGCTTATTCTTGCCTCAAACCTTTCG
	B-NT4187LF-RP	GCAGCCTGAATGGCGAATGGACGCGCTGTGTGAAACCCGGTAAGTCAAATAAA
	C-NT4187RF-LP	CAGGAGCGGGTGCGGGTGCTGGAGCGATGGCCGACTACGATCGCAGA
	D-NT4187RF-RP	AACAGCGACATCCTCTGGCTTCC
	E-NT4187-Nest-LP	TGTGGGGCATTTGGGTGTTGTAA
	F-NT4187-Nest-RP	CGGCAACAACTGTCTGAGGGATG
yfpn-sad-8 construction	SAD-8-E	AACACAACTCTTTCGGGGCGATT
	SAD-8-NGFP1	GCAGCCTGAATGGCGAATGGACGCGCTGATGTCAATGGCGATGGTAGACAG
	SAD-8-NGFP2	CAGGAGCGGGTGCGGGTGCTGGAGCGATGGATTTAGATATTGAGATGGACGTGGA
	SAD-8-F	GGTTCGCAAGCGTTGAAAAGACC
	SAD-8-G	ATAGACGGGCGAGGCGTGTGTC
	SAD-8-H	AATCCATCCGTGCGTCACTATCA
yfpn-sms-2 construction	SMS-2-E	GTCCACTTGGTGCCATTCCCACT
	SMS-2-NGFP1	GCAGCCTGAATGGCGAATGGACGCGCGCGGAGGGTGTCAAAACTCACAA
	SMS-2-NGFP2	CAGGAGCGGGTGCGGGTGCTGGAGCGATGTCTGCTCCTGGCTCTCCC
	SMS-2-F	GTGCCATTCTGCTGCTTCCAGTT
	SMS-2-G	CACTTGCCTACCACGCCATGATT
	SMS-2-H	TGCTCAAACCGCCGTAATTGTTG

Primers for DJ-PCR-based fluorescent tagging were designed as previously described [21,35].
